# MASCC/ISOO Clinical Practice Statement: Management of oral manifestations of chronic graft-versus-host-disease

**DOI:** 10.1007/s00520-024-08686-x

**Published:** 2024-07-25

**Authors:** Yehuda Zadik, Judith E. Raber-Durlacher, Joel B. Epstein, Alessandra Majorana, Alexa Laheij, Elena Bardellini, Nicole Blijlevens, Sharon Elad

**Affiliations:** 1 Department of Oral Medicine, Faculty of Dental Medicine, The Hebrew University of Jerusalem, and Hadassah-University Medical Center, P.O.B. 12272, 9112002 Jerusalem, Israel; 2 Saligman Clinics, Faculty of Dental Medicine, The Hebrew University of Jerusalem, and Hadassah-University Medical Center, Jerusalem, Israel; 3 Department of Oral Medicine, Academic Center for Dentistry Amsterdam (ACTA), University of Amsterdam and VU University, Amsterdam, The Netherlands; 4 Department of Oral and Maxillofacial Surgery, Amsterdam UMC, University of Amsterdam, Amsterdam, The Netherlands; 5 Cedars-Sinai Health System, Los Angeles, CA USA; 6 Dental Oncology Service, City of Hope Comprehensive Cancer Center, Duarte, CA USA; 7 Department of Medical and Surgical Specialities, Radiological Sciences and Public Health, School of Pediatric Dentistry, University of Brescia, Brescia, Italy; 8 Department of Hematology, Radboud University Medical Center, Radboud Institute for Health Sciences, Nijmegen, The Netherlands; 9 Oral Medicine, Eastman Institute for Oral Health, University of Rochester Medical Center, Rochester, NY USA

**Keywords:** Bone marrow transplantation, Cancer, Graft-versus-host-disease, Hematopoietic stem cell transplantation, Oral mucosa, Oral manifestations, Salivary glands

## Abstract

**Purpose:**

A MASCC/ISOO Clinical Practice Statement (CPS) is aimed at generating a concise tool for clinicians, which concentrates practical information needed for the management of oral complications of cancer patients. This CPS is focused on the management of oral manifestations of chronic graft-versus-host-disease (cGVHD).

**Methods:**

This CPS was developed based on critical evaluation of the literature followed by a structured discussion of a group of leading experts, members of the Oral Care Study Group of MASCC/ISOO. The information is presented in the form of succinct bullets and table to generate a short manual about the best standard of care.

**Results:**

The treatment goals in oral cGVHD are to relieve pain and xerostomia, improve oral function, prevent secondary infection, prevent deterioration of the dentition, and detect malignant transformation as early as possible. The prevention and treatment measures for oral mucosal lesions, hypofunction of the salivary glands, and sclerodermatous changes in the oral and perioral tissues are detailed, as well as the possible complications and side effects of these interventions.

**Conclusions:**

Patients post allogeneic hematopoietic cell transplantations, with cGVHD manifest in the oral and perioral tissues, should be regularly monitored and treated as needed by an oral care practitioner. This CPS provides the clinician with practical tools for examining, preventing, and treating the various sequalae that may affect the oral cavity in these patients.

**Supplementary Information:**

The online version contains supplementary material available at 10.1007/s00520-024-08686-x.

## Introduction

Chronic graft-versus-host disease (cGVHD) is a multi-system immune-mediated disease that may develop in patients post allogeneic hematopoietic cell transplantation (HCT). Its oral manifestations mimic auto-immune diseases such as lichen planus, Sjögren syndrome, and scleroderma. Oral and/or perioral tissues can be severely affected, causing pain, dry mouth, trismus, limited oral function, impaired quality of life, and an increased risk of oral dysplasia and cancer [1–3].

The main oral and perioral manifestations of cGVHD, which may appear in combination or as a single condition, include:

- *Mucosal lesions*. These are usually lichenoid lesions, with or without erosion and ulceration, and desquamative gingivitis. Occasionally, the lesion may have a white plaque form, which may be single or multi-focal, with or without the typical lichenoid clinical appearance. These lesions may be associated with pain and sensitivity. They may harbor a dysplastic change, although it is not trivial to identify these clinically.
- *Salivary gland disease*. This Sjögren-like salivary dysfunction may impair saliva secretion, both quantitatively and qualitatively, affecting nutrition and quality of life. Indirectly, the risk of rampant dental caries and oral candidiasis is increased.
- *Scleroderma*. This is a slow-progressing process that may eventually lead to fibrosis and soft tissue dysfunction (e.g., the tongue). Perioral sclerodermatous changes may restrict mouth opening, limit oral intake, and cause dysphagia. The perioral sclerosis may also cause difficulty sealing the lips, which may also contribute to dysphagia. The limited mouth opening and loss of flexibility of tissue compromise oral hygiene, which increases the risk of gingivitis and dental caries, and make dental care difficult. Focal gingival recession may be a manifestation of this phenomenon.
- *Oral squamous cell carcinoma (SCC)*. This post-transplant malignancy affects cGVHD patients more frequently than non-cGVHD patients, probably because of the combination of extended tissue inflammation and immunosuppression. It affects mostly the tongue, buccal mucosae, and gingival tissues. About one-quarter to one-half of the oral SCC patients present with recurrent or second primary carcinomas.
- *Other oral lesions*. Superficial mucoceles may present either as a single lesion or in clusters. Less commonly, verruciform xanthomas and pyogenic granulomas are seen.
- *Taste change (dysgeusia)*. Taste change often affects patients post allogeneic HCT. It remains unclear if this phenomenon is related directly to oral cGVHD.

Due to the complexity of multi-tissue involvement, the clinician should be familiar with the specific oral care needs of cGVHD patients and plan the treatment accordingly. It has been a decade since the US National Institutes of Health (NIH) released its guidelines for the management of oral cGVHD, and there have been new publications [4]. Therefore, a working group of the Oral Care Study Group (OCSG) of the Multinational Association of Supportive Care in Cancer (MASCC) and the International Society of Oral Oncology (ISOO) composed an expert-opinion Clinical Practice Statement (CPS) in order to provide a concise summary of the approach to oral cGVHD. In the absence of formal MASCC/ISOO guidelines, this CPS is the optimal road map to the management of oral manifestations of cGVHD. The Statement does not refer to non-cGVHD-related oral manifestations in the post-transplant patient, such as post-transplant lymphoproliferative disorders.

## Objective

To develop a practical tool for clinicians that briefly outlines the best standards of care for the various oral manifestations of cGVHD.

## Methods

Following the MASCC/ISOO Guidelines Policy, a critical evaluation of the literature using MEDLINE database searches for English language articles was conducted by a group of leading experts. The search focused on clinical trials and large case series about cGVHD-related oral mucosal, sclerodermatous, or salivary manifestations. During the development of the manuscript, point questions that deemed a closer look were generated, and a literature search was done to ensure accuracy of information. The draft was then discussed in a multi-step process by an international working group of the Oral Care Study Group of MASCC/ISOO and approved by two independent boards: the ISOO Advisory Board and the MASCC Guidelines Committee.

## Management

- As cGVHD is a systemic disease, the overall management goal is to control the disease, to maintain optimal function, and to preserve adequate quality of life. Organ involvement with cGVHD determines the specific management goals. Below is a description of the aspects pertinent to oral cGVHD.
- The treatment goals in oral cGVHD are to relieve pain and xerostomia, improve oral function (e.g., eating, mouth opening), prevent secondary infection, prevent deterioration of the dentition, and detect malignant transformation as early as possible. Overall, this will improve quality of life and possibly life expectancy.
- Patients post allogeneic HCT, who are at risk for cGVHD, should be examined by an oral health care professional every 6–12 month for oral cGVHD manifestations, or sooner if the patient notices any changes. Biopsy and pathological examination should be performed on lesions suspicious for oral cancer [5].
- Generally, cGVHD is treated with systemic corticosteroids and steroid-sparing immunomodulators, including the classic calcineurin inhibitors as well as the more recent mechanism-based targeted agents. However, when the oral mucosa is the only site of cGVHD involvement or when the oral cGVHD is resistant to systemic treatment, topical treatment is of outmost importance. In such cases, the following treatment principles are suggested:
  - When the lesions are generalized, a rinse may be more practical, whereas a gel may be more applicable for localized lesions. For gingival involvement, application in a medication tray or as a gauze occlusion may facilitate longer contact time.
  - Topical corticosteroids are the first line of therapy (Table 1). The type of preparation and its concentration depend on the extent and severity of the oral lesions. When the lesions are ulcerative/erosive and symptomatic, an ultra-potent steroid is preferred. The topical steroid selection is also tailored to the patient’s tolerance of its consistency and mode of application. It is recommended to avoid long-term application of steroid on the lips. Although topical application uncommonly causes systemic adverse effects, clinicians should be aware of this potential risk [6]. This is mostly applicable for patients with ulcerative oral cGVHD who use topical steroids for longer durations. Topical application of potent steroids with lower bioavailability, such as budesonide, may be a preferred alternative.
  - Topical tacrolimus or pimecrolimus (Table 1) [7] may be considered when topical steroids have an insufficient clinical effect. Patients should be informed that the medication may cause burning during initial application of tacrolimus ointment, which usually subsides with subsequent applications.
  - For persistent, localized symptomatic oral lesions, intralesional steroid injections (Table 1) may be administered. Although some patients experience relief following a single injection, others need a few cycles of injections to achieve the desired outcome. Triamcinolone acetonide is the common steroid reported in the dental literature for intralesional injection in the management of inflammatory oral ulcers [8]. Additional optional steroids, side effects, complications, and pitfalls have been reported in the dermatology literature [9].
  - Pain management should be considered. Topical agents for pain management include local anesthetics, local anti-histamines, or other agents that were reported to alleviate pain in oral mucositis [10]. Systemic pain management should be monitored, given the chronicity of the disease and risk for adverse effects in long-term use.
  - Early evidence suggesting the benefits of phototherapy (PUVA or UVB), photobiomodulation [11, 12], and platelet gel has been published. Topical belumosudil suspension was tested for safety and tolerability in oral cGVHD patients. More research is needed about these interventions.
- Exophytic lesions, such as pyogenic granuloma and verruciform xanthoma, necessitate surgical excision followed by histopathological analysis of the excised tissue. In contrast, superficial mucoceles typically do not require a biopsy for diagnostic confirmation.
- Patients with sclerodermatous-type cGVHD are often treated systemically. In addition, these patients should be instructed to practice daily physical therapy such as active stretching of the tongue and perioral muscles, to prevent further oral and perioral fibrosis and preserve the range of motion. During follow-up appointments, the patient should be encouraged to continue performing these exercises. Other suggested treatment strategies include surgery, and intralesional corticosteroid injections.
- For detailed management protocols of hyposalivation or xerostomia, clinicians are referred to the OCSG CPS about Salivary Gland Hypofunction and Xerostomia [13]. Below are some general principles in the management of hyposalivation pertinent to cGVHD patients:
  - Pharmacological sialogogues, such as pilocarpine and cevimeline, may be considered, taking into account the contraindications and possible adverse effects [14].
  - Over-the-counter moistening agents may be used for palliation of dry mouth. Nonpharmacological saliva stimulants, such as sweet/sour sugar-free hard candies or chewing gums, may be used.
  - In refractory cases, intraductal irrigation of the parotid glands [15] or electrostimulation therapy [16] may be considered for xerostomia relief.
  - Frequent topical application of high-concentration fluoride preparation may help prevent dental caries [17]. Patient should be educated and encouraged to practice meticulous oral hygiene.
- Given that secondary oral candidiasis is relatively common in patients with hyposalivation treated with corticosteroids, antifungal treatment may be needed. Topical antifungals (e.g., nystatin, miconazole, clotrimazole) are advised for oral candidiasis. Antifungal lozenges should be avoided in cases of severe dry mouth, as such conditions do not facilitate the lozenges disintegration. Patients should be instructed to comply with the dosing protocol in order to achieve the best effect and to follow basic principles of resistance development prevention. Systemic antifungals (e.g., fluconazole) may also be considered. Denture wearers should be advised to minimize use of their prostheses (for example, removing them during sleep), and to clean, disinfect, and soak the prostheses in antifungal solution upon removal.
- The oral cGVHD manifestations may negatively impact the dentition. Hyposalivation due to cGVHD or the pretransplant high-intensity conditioning regimen may increase the risk for dental caries, and desquamative gingivitis may compromise oral hygiene practice [18, 19]. Furthermore, the limitation of mouth opening and loss of vestibular elasticity may be a barrier for oral and dental care. Therefore, the patient should be educated about the necessity of frequent dental checkups and meticulous oral hygiene tailored to their specific needs.

**Table 1:** Recommended topical immunomodulators regimens for oral mucosal chronic graft-versus-host disease (modified from Elad et al. [7])

Medication	Agents	Concentration	Daily dose
Solutions	Dexamethasone	0.1–0.5 mg/mL (0.01–0.05%)*	5 mL × 1–6/day
	Prednisolone	3 mg/mL (0.3%)	5 mL × 1–6/day
	Budesonide	0.3–0.6 mg/mL (0.03–0.06%)*	5 mL × 1–4/day
	Clobetasol	0.5 mg/mL (0.05%)*	5 mL × 1–3/day
	Triamcinolone	1 mg/mL (0.1%)*	5 mL × 1–2/day
	Triamcinolone	10 mg/mL (1%)*	5 mL × 1–6/day
	Betamethasone	0.5 mg/mL (0.05%)*	5 mL × 1–4/day
	Tacrolimus**	0.1 mg/mL (0.01%)*	5 mL × 1–6/day
Creams/gels/ointments/pastes	Clobetasol (c, g)	0.05%	× 1–2/day
	Triamcinolone (c, o, p)	0.1–0.5%	× 1–2/day
	Halobetasol (c)	0.05%	× 1–2/day
	Betamethasone (c, g, o)	0.05–0.1%	× 1–2/day
	Fluocinonide (g)	0.05%	× 1–2/day
	Tacrolimus (o)**	0.03–0.1%	× 1–3/day
	Pimecrolimus (c)**	1%	× 1–2/day
Intralesional injection	Triamcinolone	10–40 mg/mL	× 1***

*c*, cream; *g*, gel; *o*, ointment; *p*, paste

The list of topical agents was obtained from the National Institute of Health (NIH) Consensus Development Project on Criteria for Clinical Trials in Chronic Graft-versus-Host Disease: V. The 2014 Ancillary Therapy and Supportive Care Working Group Report. Cyclosporine compounded solution was excluded due to inconsistent data regarding concentrations. Some commercial steroidal preparations contain anesthetics, such as lidocaine. Availability of medications may be subject to geographic variations

*The preparation is not available commercially in the USA, and the patient should be advised about the compounding options

**The clinician should be aware of the caution note in the manufacturer information sheet (Black Box: skin malignancies and lymphoma reported following topical calcineurin inhibitor use, causal relationship not established; avoid continuous long-term use). No specific data on malignant transformation following oral topical application

***Either a single injection or as a series of treatments with 3-week intervals

### Supplementary Information

Below is the link to the electronic supplementary material.

Supplementary file1 (DOCX 17.4 KB)

## Data Availability

No datasets were generated or analysed during the current study.

## Declarations

### Disclaimer

The MASCC/ISOO OCSG Statements have been developed to facilitate the expert-opinion based management of oral complications of cancer therapy where high-quality evidence is lacking. Clinicians should use their judgment when making treatment decisions for individual patients. The Statement authors and the MASCC/ISOO do not guarantee or take responsibility for clinical outcomes in individual patients.
