# Supplementary material for: MASCC/ISOO Clinical Practice Statement: Management of oral manifestations of chronic graft-versus-host-disease
Source: Support Care Cancer. 2024 Jul 25;32(8):546. doi: 10.1007/s00520-024-08686-x (PMC11269426; doi:10.1007/s00520-024-08686-x)
Supplement: Supplementary file 1 — Supplementary file1 (DOCX 17.4 KB) [file 520_2024_8686_MOESM1_ESM.docx]

**MASCC/ISOO Clinical Practice Statement: Management of oral manifestations of chronic graft-versus-host-disease**

**Suggested reading:**

1. Haas L, Cruz-Pamplona M. Topical treatment of oral chronic graft-versus-host- disease in hematopoietic stem cell transplant recipients: A systematic review. J Clin Exp Dent. 2023 May 1;15(5):e420-e427. doi: 10.4317/jced.60138. PMID: 37214749; PMCID: PMC10198688.
2. Pukhalskaya T, Smoller BR, Becker M, Maly A, Zadik Y, Elad S. Oral white lesion in patients post-hematopoietic stem cell transplantation: a case series demonstrating the diagnostic dilemma. Support Care Cancer. 2021 Dec;29(12):7999-8007. doi: 10.1007/s00520-021-06392-6. Epub 2021 Jul 4. PMID: 34218349.
3. Zadik Y. Restricted mouth opening in chronic graft-versus-host disease. Oral Surg Oral Med Oral Pathol Oral Radiol. 2016 Feb;121(2):201-2. doi: 10.1016/j.oooo.2015.07.041. Epub 2015 Oct 14. PMID: 26686955.
4. Bajonaid A, Guntaka PK, Harper M, Cutler C, Duncan C, Villa A, Sroussi HY, Woo SB, Treister NS. Characterization of orofacial features in sclerodermatous chronic graft-versus-host disease. Oral Dis. 2024 Mar 21. doi: 10.1111/odi.14932. Epub ahead of print. PMID: 38514965.
5. Elad S, Zadik Y, Zeevi I, Miyazaki A, de Figueiredo MA, Or R. Oral cancer in patients after hematopoietic stem-cell transplantation: long-term follow-up suggests an increased risk for recurrence. Transplantation. 2010 Dec 15;90(11):1243-4. doi: 10.1097/TP.0b013e3181f9caaa. PMID: 21119507.
6. Bar O, Elad S, Avni B, Abu-Tair J, Zaharia B, Hanut A, Zadik Y. Oral verruciform xanthoma in chronic graft-versus-host disease patients. Support Care Cancer. 2021 Jan;29(1):79-84. doi: 10.1007/s00520-020-05681-w. Epub 2020 Aug 15. PMID: 32803727.
7. Zadik Y, Elad S, Shapira A, Shapira MY. Treatment of oral mucosal manifestations of chronic graft-versus-host disease: dexamethasone vs. budesonide. Expert Opin Pharmacother. 2017 Feb;18(3):235-242. doi: 10.1080/14656566.2017.1282464. Epub 2017 Jan 25. PMID: 28081677.
8. Bulthuis MS, van Leeuwen SJM, Thomas RZ, van Gennip LLA, Whiteside HM, Isom S, Kline DM, Laheij AMGA, Raber-Durlacher JE, Hasséus B, Johansson JE, Hovan AJ, Brennan MT, von Bültzingslöwen I, Huysmans MDNJM, Blijlevens NMA. Subjective Oral Dryness following Hematopoietic Cell Transplantation: A Report from the Orastem Study. Transplant Cell Ther. 2024 Apr;30(4):446.e1-446.e11. doi: 10.1016/j.jtct.2024.01.067. Epub 2024 Jan 17. PMID: 38242439.
9. Elad S, Or R, Shapira MY, Haviv A, Galili D, Garfunkel AA, Bitan M, Kaufman E. CO2 laser in oral graft-versus-host disease: a pilot study. Bone Marrow Transplant. 2003 Nov;32(10):1031-4. doi: 10.1038/sj.bmt.1704272. PMID: 14595392.
10. Schueller O, Regev G, Singh N, Willson A, Beville M, Kanji N, Lohmer L, Patel J. Two-Part Phase 1 Study to Evaluate the Taste Profile of Novel Belumosudil Oral Suspensions and Assess the Relative Bioavailability and Food Effect of the Selected Belumosudil Oral Suspension Compared With Oral Tablet Reference in Healthy Male Participants. Clin Pharmacol Drug Dev. 2024 May;13(5):491-498. doi: 10.1002/cpdd.1378. Epub 2024 Feb 12. PMID: 38345529.
